# Balancing the Encoder and Decoder Complexity in Image Compression for Classification

**DOI:** 10.21203/rs.3.rs-4002168/v1

**Published:** 2024-04-22

**Authors:** Zhihao Duan, Adnan Faisal Hossain, Jiangpeng He, Fengqing Zhu

**Affiliations:** Elmore Family School of Electrical and Computer Engineering, Purdue University, West Lafayette, 47907, IN, U.S.A.

**Keywords:** Coding for machines, rate-accuracy-complexity, learned image compression

## Abstract

This paper presents a study on the computational complexity of coding for machines, with a focus on image coding for classification. We first conduct a comprehensive set of experiments to analyze the size of the encoder (which encodes images to bitstreams), the size of the decoder (which decodes bitstreams and predicts class labels), and their impact on the rate-accuracy trade-off in compression for classification. Through empirical investigation, we demonstrate a complementary relationship between the encoder size and the decoder size, i.e., it is better to employ a large encoder with a small decoder and vice versa. Motivated by this relationship, we introduce a feature compression-based method for efficient image compression for classification. By compressing features at various layers of a neural network-based image classification model, our method achieves adjustable rate, accuracy, and encoder (or decoder) size using a single model. Experimental results on ImageNet classification show that our method achieves competitive results with existing methods while being much more flexible. The code will be made publicly available.

## 1 Introduction

Data compression is a fundamental problem in information theory as well as many real-world applications. Recently, *coding for machines* emerges as a promising scheme in data compression, in which case one aims to represent data using as few bits as possible while retaining high prediction accuracy for downstream vision tasks. Coding for machine approaches have already many potential applications such as edge-cloud computing [1–3], privacy-preserving communication [4, 5], and the Internet of Things [6, 7].

Most existing research on coding for machines focuses on the *rate-accuracy* trade-off, where *rate* measures the average number of bits per sample produced by the encoder, and *accuracy* measures the downstream task (e.g., ImageNet classification [8]) prediction accuracy on the decoder side. This is a natural extension of the classical *rate-distortion* trade-off in lossy compression, and various prior works have studied coding for machine problems based on rate-distortion theory [9–11]. In particular, Dubois et al. [9] have theoretically proved that, for certain tasks, it is possible to achieve a high prediction accuracy with an extremely low rate compared to reconstruction-oriented compression.

Yet what is feasible in theory may not always be achievable in practice. Rate-distortion theory, which describes the best possible rate-distortion (or rate-accuracy in our context) trade-off, assumes an unbounded encoder and decoder family. However, many practical applications involve constraints on computational resources (e.g., for mobile and wearable devices), which limit the choice of encoder and decoder architectures. Such constraints render the theoretical rate-accuracy bounds inapplicable, calling for a more practical analysis of coding for machine performance under computational constraints.

Motivated by this, we first study the rate-accuray trade-off in coding for machines under computational constraints on the encoder and decoder. Our analysis reveals that such constraints would greatly impair the rate-accuracy trade-off, leading to a three-way trade-off between rate, accuracy, and computational complexity. Targeting this trade-off, we propose an end-to-end learning framework for adjusting these three quantities using a single neural network model, which has not been achieved by existing methods.

To summarize, we make the following contributions to the study of coding for machines: (Sec. 3) We empirically investigate the impact of encoder/decoder size on coding for machine performance. Our analysis reveals a complementary relationship between the encoder and decoder sizes, as well as a three-way trade-off between rate, distortion, and encoding complexity; (Sec. 4 and 5) We propose a novel method for adjusting this three-way trade-off that requires only a single neural network model. Experiments show that our method achieves comparable rate-distortion-complexity performance with existing methods while being much more flexible for deployment.

## 2 Preliminaries

This section briefly reviews the theoretical foundations of lossy compression. Readers familiar with rate-distortion theory and coding for machines may skip this section.

### Rate-distortion theory.

**Fig. 1:**
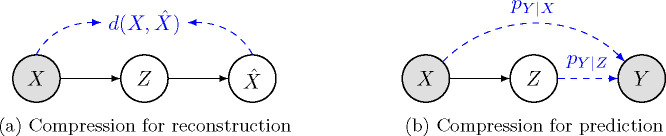
Difference between coding for human (or compression for reconstruction, Fig. 1a) and coding for machines (or compression for prediction, Fig. 1b). This paper focuses on coding for machines, where one wants to encode data X to a compressed representation Z such that predicting a target Y from Z is as good as predicting from X.

Let $X∼p_{X}$ denote the source data variable. In lossy compression for reconstruction (Fig. 1a), also referred to as *coding for human vision*, the goal is to represent X using as few bits as possible, from which one can obtain a good reconstruction $\overset{ˆ}{X}$. The distortion is quantified by a function d(⋅) measuring the difference between X and $\overset{ˆ}{X}$. Given a distortion threshold D∈R, the minimum rate (i.e., average number of bits per sample when compressing an i.i.d. sequence of X) required to achieve $E[d(X,\overset{ˆ}{X})]\leq D$ is given by the information rate-distortion function [12]:

(1) $$R\left(D\right)=\underset{p_{\overset{ˆ}{X}|X}}{min}\;I(X;\overset{ˆ}{X})\;\text{s.t.}\;E\left[d(X,\overset{ˆ}{X})\right]\leq D,$$

where I(⋅) is mutual information. Note that the reconstruction $\overset{ˆ}{X}$ need not be in the same space as the source X. In coding for machines, for example, the reconstruction is often a prediction target instead of the original data.

### Rate-distortion in coding for machines.

Let $p_{X,Y}$ be a joint distribution, where X denotes data, and Y is the prediction target. In compression for prediction (Fig. 1b), we again want to represent X using a low-rate representation, but on the decoding side, the objective now is to infer Y instead of reconstructing X. Prior research has applied the rate-distortion theory to coding for machines in various ways [9–11]. Among them, a representative approach [9] is to regard the compressed representation Z as the “reconstruction” and adopt the following distortion function:

(2) $$d\left(X,\;Z\right)≜H\left(Y|Z\right)-H\left(Y|X\right)=D_{KL}\left(p_{Y|X}∥p_{Y|Z}\right),$$

where $D_{KL}$ is the KL divergence. Intuitively, Eq. (2) measures how well a representation z can be used to predict Y, compared to predicting Y using x. This distortion equals the best-case classification log loss and simulates the prediction error in many downstream tasks. With this distortion, the information rate-distortion function becomes:

(3) $$R\left(D\right)=max\left(I\left(X;Y\right)-D,0\right),$$

which follows from Theorem 2 of [9]. Intuitively, the distortion threshold D determines the maximum information loss regarding Y allowed during encoding. When D=0, no information loss is allowed, and the encoder must retain all task-related information I(X;Y) in the representation Z. In this case, the minimum achievable rate equals I(X;Y), and the compression is lossless in the sense that predicting from Z is as good as predicting from X. When D>0, the encoder is allowed to discard a subset of I(X;Y), and the minimum achievable rate decreases accordingly.

### Assumptions.

We consider neural network-based (as opposed to hand-crafted) encoders and decoders, the complexity of which can be controlled by tuning the number of network layers and the number of dimensions per layer. We apply elementwise uniform quantization and use factorized entropy models [13] for Z. We consider image classification as the downstream prediction task. More general settings (e.g., vector quantization and other downstream tasks) are left to future work.

## 3 Rate-Distortion under Computational Constraints

We perform two experiments (Sec. 3.1 and Sec. 3.2) to study the impact of encoder and decoder size on the R-D trade-off in coding for machines. In both experiments, we assume that the encoder and decoder are neural networks accompanied by uniform scalar quantization, following the *non-linear transform coding* framework [14]. Computational constraints are thus imposed by varying the neural network depth (number of layers) and width (number of dimensions per layer). Sec. 3.3 discusses the observations and motivates our proposed method for image coding for machines (Sec. 4).

### 3.1 Experiment: 2-D datapoint classification

**Fig. 2:**
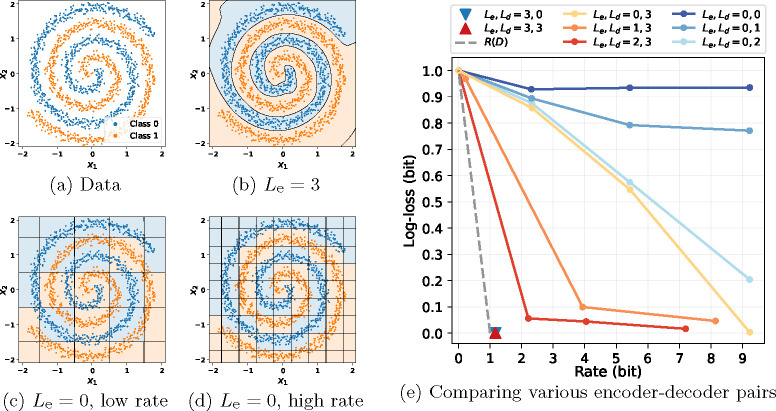
We train neural compressors with various encoder/decoder sizes to compress the data points in (a) for classification. The encoder and the decoder are MLPs with $L_{e}$ and $L_{d}$ hidden layers, respectively. $L_{e}=0$ refers to a elementwise uniform quantizer. Figures (b), (c), and (d) show the quantization boundaries of the encoders, where the background colors indicate the predicted label. Figure (e) shows the rate-distortion results for various encoder-decoder pairs.

We considers a toy problem of 2-D datapoint compression for classification, shown in Fig. 2a. The data X is 2-D, the label Y is binary with equal probability, and the difference between the two classes is an angular shift in polar coordinates. In this experiment, the rate is measured by bits per datapoint, and the distortion is measured by classification log loss (also known as the cross-entropy loss).

According to Eq. (3), we know that one could achieve no distortion with R(0)=I(X;Y)=H(Y)=1 bit per datapoint (when compressing long sequences). To verify this, we train a neural compressor [14] with a powerful encoder and decoder, both of which are MLPs with 3 hidden layers. Its R-D performance is shown as the red triangle marker in Fig. 2e. We observe that this particular compressor successfully achieves a performance close to R(0), i.e., zero distortion with a rate close to 1 bit.

Then, we reduce the encoder and decoder sizes by decreasing the number of hidden layers and dimensions. Let $L_{e}$ denote the number of hidden layers in the encoder, and $L_{d}$ the number of hidden layers in the decoder. We try with various combinations of $L_{e}\in [0,\;3]$, $L_{d}\in [0,\;3]$ and show the results in Fig. 2e. We make the following observations based on the results:

- **Given a powerful encoder, restricting the decoder does not hurt R-D:** we keep $L_{e}=3$ and reduce $L_{d}$ to 0 (i.e., a linear decoder). This configuration is shown as the blue triangle mark in Fig. 2e. In this case, restricting the decoder size does not affect the R-D performance, indicating that given a powerful encoder, a simple decoder suffices to make accurate predictions.
- **Given a powerful decoder, restricting the encoder increases rate:** we gradually reduce $L_{e}$ from 3 to 0 while keeping $L_{d}=3$. Note that $L_{e}=0$ refers to a simple elementwise uniform quantizer. Results are shown as the yellow and red curves in Fig. 2e. We see that to achieve the same distortion, the rate increases as the encoder size decreases. Note that even weak encoders are able to achieve near-zero distortion, as long as given a sufficiently high rate (e.g., when $L_{e}=0$, $L_{d}=3$).
- **For a weak encoder, restricting the decoder increases distortion:** we keep $L_{e}=0$ and decrease $L_{d}$ from 2 to 0, shown as the blue curves in Fig. 2e. This increases the distortion at all rates, indicating that a powerful decoder is necessary to make accurate predictions when using weak encoders.

To better understand the behavior of the encoders with different number of layers, we visualize their quantization regions in Fig. 2b for $L_{e}=3$, and Fig. 2c and 2d for $L_{e}=0$. We see that the large encoder (Fig. 2b) encodes only task-related information, which is the polar angular shift in this toy problem, and data points with the same label are quantized to the same code. In contrast, the small encoder (Fig. 2c and 2d) is not able to extract the task information due to its restricted capacity, and extra bits are used to code task-irrelevant information, i.e., datapoint positions in Cartesian coordinates.

This toy problem of 2-D classification gives intuitions about the functionality of the encoder and decoder with different sizes (and thus different expressive power). Next, we conduct experiments for natural images to verify our observations.

### 3.2 Experiment: CIFAR-10 classification

**Fig. 3:**
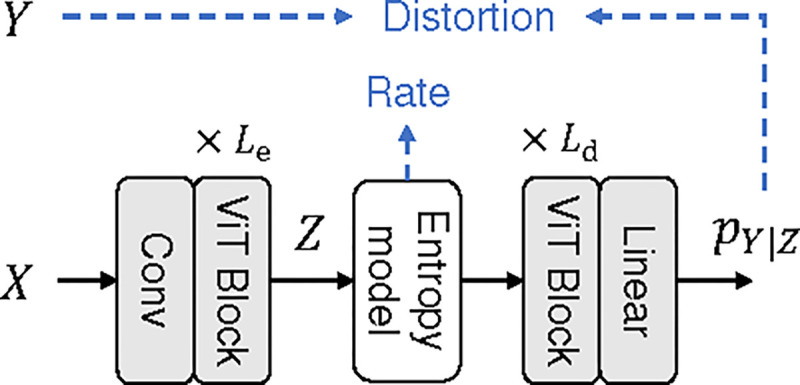
Vision transformer (ViT)-based compressor.

**Fig. 4:**
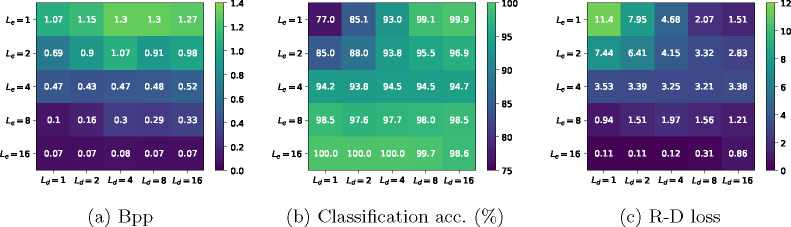
CIFAR-10 compression for classification results. We train the model (Fig. 3) with various combinations of encoder layers $L_{e}$ and decoder layers $L_{d}$, and we report the bits per pixel (bpp), classification accuracy, and R-D loss for each of them. Lower bpp and R-D loss are better, and higher classification accuracy is better.

This section considers a more realistic scenario where we use vision transformer-based [15] encoders and decoders to compress the CIFAR-10 dataset for classification. CIFAR-10 [16] is a natural image classification dataset that is widely used in machine learning research. It contains 60,000 RGB images (50,000 for training, 10,000 for testing) of ten different object categories, and each image has 32 × 32 pixels. We use a vision transformer-based model (shown in Fig. 3) to compress the images and make predictions from the compressed representation. The encoder and decoder sizes are controlled by their number of ViT blocks, denoted by $L_{e}$ and $L_{d}$, respectively. Rate is measured by bits per pixel (bpp), distortion is measured by the cross-entropy loss, and the Lagrange multiplier used is λ=16.0. We train the model for 100k iterations with batch size 256, learning rate 0.001, Adam optimizer [17], and cosine learning rate schedule. We apply various combinations of $L_{e}$ and $L_{d}$, and the experimental results are presented in Fig. 4. The main observations are summarized as follows.

#### Increasing the encoder size significantly reduces bpp, but increasing the decoder size does not.

This can be observed in Fig. 4a. For a fixed encoder size $L_{e}$, increasing the decoder size $L_{d}$ leads to an unchanged (for $L_{e}=16$) or worse bpp (e.g., for $L_{e}=1$). However, for a fixed $L_{d}$, increasing $L_{e}$ from 1 to 16 reduces bpp from around 1.0 to around 0.07. In other words, the rate is highly (negatively) correlated with $L_{e}$, but it is approximately independent of $L_{d}$. This is largely expected, as the rate is independent of the decoder when given the data source and the encoder^1^.

#### Increasing either the encoder size or the decoder size improves the classification accuracy.

This can be seen from Fig. 4b. When fixing $L_{e}=1$ (first row in the figure), increasing $L_{d}$ from 1 to 16 improves the accuracy from 77.0% to 99.9%. When fixing $L_{d}=1$ (first column in the figure), a similar trend can be observed. This suggests that a powerful end-to-end model (i.e., the encoder concatenated with the decoder) is necessary to make accurate predictions. Note that increasing the model size does not always improve classification accuracy. For example, when $L_{e}=16$, increasing the decoder size $L_{d}$ from 4 to 16 decreases the accuracy from 100.0% to 98.6%. This is presumably because training larger models typically requires more training iterations and data [18], which is not the case in our simple setting (we use the same training recipe for all models).

#### A complementary relationship exists between the encoder and decoder sizes.

Looking at Fig. 4c, we see that the best choice of $L_{d}$ for $L_{e}=\;1$ is $L_{d}=16$, indicating that a powerful decoder is necessary to achieve good R-D performance when the encoder is weak. Contrarily, $L_{d}=1$ is one of the best choices when $L_{e}=16$, indicating that a simple decoder is sufficient when the encoder is powerful. If we list the best $\left(L_{e},L_{d}\right)$ pairs for each $L_{e}$ (i.e., we compute *argmin* for the rows in Fig. 4c), we obtain $\left(L_{e}=1,L_{d}=16\right)$, $\left(L_{e}=2,L_{d}=16\right)$, $\left(L_{e}=4,L_{d}=8\right)$, $\left(L_{e}=8,L_{d}=1\right)$, $\left(L_{e}=16,L_{d}=\right.1\;or\;2)$. That is, as the encoder size increases, the best choice of the decoder size decreases.

### 3.3 Desired properties of a practical method

Our observations in previous sections suggest several insights for designing a flexible and efficient method for compression for prediction. First, since there is a multi-way trade-off among rate, distortion, and encoder capacity, a flexible method should be capable of operating at various combinations of these factors to accommodate different application scenarios. Second, the method may take advantage of the complementary relationship between the encoder and decoder. For example, when the encoder is powerful enough, one needs only a simple decoder to produce an accurate prediction. Otherwise, a complex and powerful decoder is needed. In the following section, we propose a method that satisfies these properties.

## 4 Adjusting the Rate, Prediction Accuracy, and Encoder-Decoder Complexity Using a Single Model

We present an end-to-end framework for image compression for prediction. Our method, FICoP (**F**lexible **I**mage **Co**mpression for **P**rediction), uses only a single model to achieve adjustable rate, distortion, and encoding/decoding complexity, making it a flexible method for practical applications.

### 4.1 Overview

**Fig. 5:**
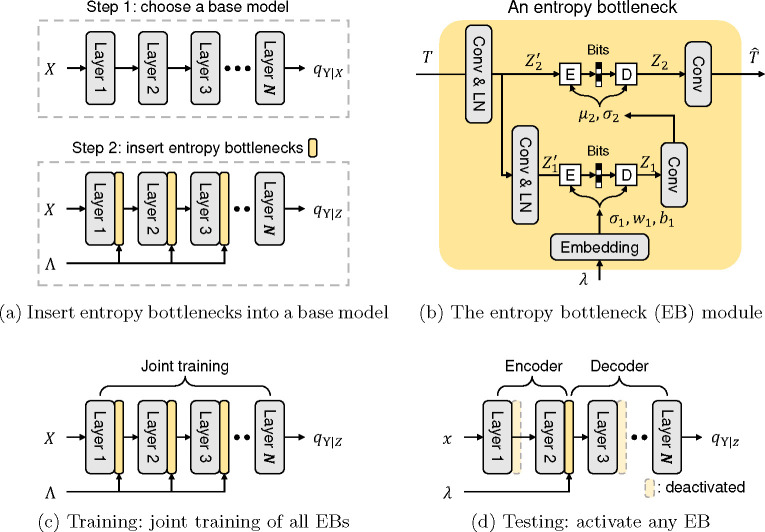
Overview of our method. In figure (b), 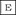 denotes entropy coding (with quantization), 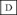 denotes decoding, and LN denotes Layer Normalization.

Fig. 5 overviews our approach. Suppose an existing neural network model takes data X as input, produces a (first-order) Markov chain of features, and predicts the conditional label distribution $q_{Y|X}$ (we use q to distinguish it from the true data distribution $p_{X,Y}=p_{X}\cdot p_{Y|X}$). Our method is a plug-and-play extension that takes the base model and inserts entropy bottlenecks (EBs) in between its layers (Fig. 5a). Each EB can be viewed as a splitting point that divides the model into an encoder $q_{Z|X}$ and a decoder $q_{Y|Z}$, and at test time (Fig. 5d), one can freely choose which EB to activate. By splitting the model in such a way, we can control the encoding complexity and, at the same time, take care of the complementary relationship between the encoder and decoder. For example, activating an EB at the early layer results in a small encoder and a large decoder, and vice versa.

For each EB, the training objective is to minimize the rate-distortion Lagrangian:

(4) $$\underset{q_{Z|X},q_{Y|Z}}{min}\;\lambda \cdot H\left(Z\right)+E_{X∼p_{X},Z∼q_{Z|X}}\left[D_{KL}\left(p_{Y|X}∥q_{Y|Z}\right)\right],$$

where H(Z) denotes the entropy of the discrete latent representation Z estimated by a neural entropy model [13], the KL term is the distortion, and λ is a Lagrange multiplier that trades off rate and distortion. A key contribution in our method is to optimize Eq. 4 for multiple EBs at a range of λ using a single end-to-end training process (Fig. 5c). Note that we use λ in the loss function as well as pass it to the model as an input.

### 4.2 The entropy bottleneck (EB) module

Fig. 5b shows the entropy bottleneck module. It follows the Hyperprior structure [13], a two-layer VAE architecture commonly used for image compression. The compressed representation Z contains two components $Z_{1}$ and $Z_{2}$ with an auto-regressive prior $p_{Z}=p_{Z_{2}|Z_{1}}\cdot p_{Z_{1}}$. We describe the details and highlight the differences from the original design as follows.

#### A lightweight bottleneck architecture.

Unlike in Ballé et al. [13] where a stack of CNN layers is used, we use single convolutional layers for all transformations. Our objective is to keep the small size of each EB so that we can insert multiple EBs into any existing model without significantly increasing the overall model size. Non-linearity is achieved by layer normalization [19] operations, which has been shown effective in image compression [20–22]

#### Quantization with straight-through gradient estimator.

As the standard practice, we quantize $Z_{1}$ and $Z_{2}$ using elementwise uniform quantization before invoking the entropy coding algorithm. However, as opposed to the additive uniform noise in [13], we apply hard quantization during training as well, and the gradients are approximated using the straight-through estimator (STE) [23, 24]. Although STE is shown sub-optimal in compression for reconstruction [25, 26], we find that STE is better than additive uniform noise in our setting (Appendix B.2).

#### Probablistic models and variable-rate compression.

We model the prior for $Z_{1}$ and $Z_{2}$ using the discretized Gaussian distribution [27]:

(5) $$p_{Z_{1}}(z)=\int_{z-0.5}^{z+0.5}\;𝒩\left(t;\;0,\sigma_{1}^{2}\right)\;dt,\;p_{Z_{2}|Z_{1}}(z)=\int_{z-0.5}^{z+0.5}\;𝒩\left(t;\mu_{2},\sigma_{2}^{2}\right)\;dt,$$

where $\sigma_{1}$ is a function of λ (through an embedding layer), and $\left\{\mu_{2},\sigma_{2}\right\}$ are functions of $Z_{1}$ (through a convolutional layer), as shown in Fig. 5b. The embedding layer consists of a sinusoidal positional encoding [28] followed by an MLP, following [29]. We achieve rate-adaptive quantization [30, 31] by applying an affine transform and its inverse to $Z_{1}$ before and after nearest integer rounding, respectively:

(6) $$Z_{1}=\mathrm{Round}\left(\frac{Z_{1}^{\prime }\;-\;b_{1}}{w_{1}}\right)\cdot w_{1}+b_{1},$$

where $Z_{1}^{\prime }$ is the variable before quantization, and the affine parameters are produced by the λ embedding layer. This adaptive quantization is also applied for $Z_{2}$, which we omit in Fig. 5b for simplicity. Equation (5) effectively conditions the prior $p_{Z}=p_{Z_{2}|Z_{1}}\cdot p_{Z_{1}}$ on λ, and Eq. (6) conditions the encoder $q_{Z|X}$ on λ, allowing us to control the rate-distortion by varying the λ input to the model.

### 4.3 Training objective

Given a base model employed with multiple EBs, we train them jointly in a single training process (Fig. 5c). Specifically, let k denote the index for the EBs, and $p_{K}$ be a pre-defined distribution over the indices, which we choose to be uniform in our experiments. At each training iteration, we activate the *k*-th EB and deactivate the others for a k sampled from $p_{K}$. To achieve variable-rate training, we also randomly sample λ from a distribution $p_{\Lambda }$ throughout training. This λ is then used in the loss function as well as to condition the EBs. In our experiments, we choose $p_{\Lambda }$ to be a log-uniform distribution with a range of [0.1,16], and we discuss these choices of hyperparameters in Appendix B.1.

Formally, the training objective is to minimize the following loss w.r.t. all model parameters (including the base model and all EBs):

(7) $$ℒ=E_{p_{X,Y},p_{K},p_{\Lambda }}\left[\Lambda \cdot log\frac{1}{p_{Z^{\left(K\right)}}(Z^{\left(K\right)})}+log\frac{1}{q_{Y|Z^{\left(K\right)}}(Y|Z^{\left(K\right)})}\right],$$

where the first term corresponds to rate, and the second corresponds to distortion.

## 5 Experiments

Without otherwise specified, we use the ResNet-50 [32] as the base model in this section. We show that our method generalizes well to other model architectures in Appendix B.

### Dataset and metrics.

We use the 1,000-class ImageNet dataset [8] for training (*train* split) and evalutation (*val* split). In evaluation, all images are resized to 224 × 224 pixels, and the rate is measured in terms of bits per pixel (bpp) after resizing. The distortion is estimated by the log loss (also known as the cross-entropy loss) of the model prediction w.r.t. the ground truth label. We also report the top-1 classification accuracy, which is more interpretable. All metrics are computed for each image in the *val* set and then averaged across the entire *val* set.

### Augment ResNet-50 with FICoP.

**Fig. 6:**
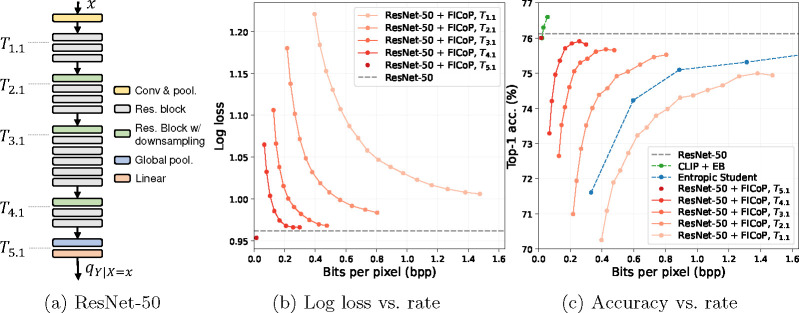
ImageNet results using ResNet-50 as a base model in terms of (b) distortion-rate and (c) accuracy-rate performance. Note that our approach (ResNet-50 + FICoP) is able to control the rate-distortion-complexity at test time using a single trained model (by activating different EBs and adjusting λ), while the baseline methods train a separate model for each R-D point.

ResNet-50 contains five stages (excluding the first convolutional layer), each of which contains multiple blocks. We take ResNet-50 with pre-trained weights as the base model and insert EBs to the splitting points shown in Fig. 6a. The splitting points are referred to as $T_{i.j}$, where i is the stage index, and j is the block index in the stage (the indices start from 1). The resulting model is referred to as *ResNet-50 + FICoP* and is trained on ImageNet *train* split for 160k iterations with a batch size of 256. For $T_{1.1}$ to $T_{4.1}$, we train the EBs with λ∈[0.1,16]. For $T_{5.1}$, we found that the model is not sensitive to λ and the rate is always close to zero, so we only train it at λ=0.1. The full details of training hyperparameters are given in Appendix A.

### 5.1 Validating the rate-distortion-complexity trade-off

Fig. 6b shows the rate-distortion (R-D) results of ResNet-50 + FICoP operating at each splitting point, and each of them produces a separate R-D curve. The encoding complexity for the case of all splitting points is reported in Table 1. We also show results for the original ResNet-50 as a reference.

Comparing the R-D curves in Fig. 6b, we observe that a deeper splitting point (which consumes higher encoding complexity) achieves better R-D performance. This is expected by our analysis, as a more powerful encoder is able to compress away more task-irrelevant information, thus reducing the rate for the same distortion. Note that when the encoding complexity approaches that of the entire ResNet-50, i.e., at $T_{5.1}$, the log loss converges to the one of the original ResNet-50 with a near-zero rate, which can be viewed as approaching the information R(D) function.

### 5.2 Comparing ResNet-50 + FICoP with existing methods

#### Existing methods.

We consider several related works as baselines. To our best knowledge, no existing method is able to achieve a rate-distortion-complexity trade-off using a single model as in ours, so a strictly fair comparison is not possible. We briefly describe these approaches as follows:

- Dubois et al. [9] uses CLIP [33] together with an entropy bottleneck as the encoder and an MLP as the decoder. We refer to this method as *CLIP + EB*. Its setting differs from ours in that (a) CLIP is trained on image-text pairs instead of ImageNet, and (b) the method operates at only high encoding complexity.
- Matsubara et al. [3] uses a lightweight CNN encoder and a trunacted ResNet-50 decoder with knowledge distillation techniques applied during training. The method is referred to as *Entropic Student*. In their setting, one needs to train multiple models to operate at different rates, and only low encoding complexity is supported.

Fig. 6c shows the accuracy-rate results of our method compared to the baseline methods, and Table 1 shows the corresponding encoding complexities. The Entropic Student method employs a lightweight encoder (around 0.47 GFLOPs) and thus achieves a much lower accuracy-rate curve than CLIP + EB (around 4.4 GFLOPs). Our method, however, is able to adjust the encoding complexity to control the accuracy-rate trade-off. In the low complexity regime, ResNet-50 + FICoP at $T_{1.1}$ and $T_{2.1}$ achieves similar accuracy-rate curves as Entropic Student, and in the high complexity regime, ResNet-50 + FICoP at $T_{5.1}$ achieves a comparable accuracy-rate curve as CLIP + EB. This demonstrates the flexibility and effectiveness of our approach in controlling the rate-distortion-complexity trade-off. Note that the accuracy-rate curves are not always monotonic because the method is trained to optimize the log loss, which does not always translate into classification accuracy.

### 5.3 FICoP with various base model architectures

#### FICoP with ConvNeXt [34].

**Fig. 7:**
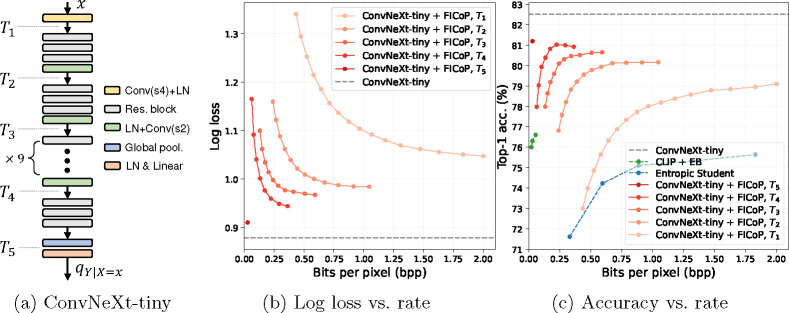
ImageNet classification using ConvNeXt-tiny as the base model in terms of (b) distortion-rate and (c) accuracy-rate performance.

To verify that our approach generalizes well to various model architectures, we choose a modern convolutional neural network architecture, ConvNeXt, as the base model. We apply FICoP to ConvNeXt-tiny, a lightweight version of ConvNeXt that achieves 82.5% top-1 accuracy on ImageNet, and show the results in Fig. 7. In the figure, Fig. 7a shows the model architecture and the layers in which we insert EBs, Fig. 7b show the distortion-rate results, and Fig. 7c show the accuracy-rate results. We observe a rate-distortion-complexity trade-off, which is consistent with our previous analysis. Furthermore, since ConvNeXt is a more powerful base model than ResNet-50, it leads to a significant performance boost when compared to the baseline methods.

#### FICoP with Swin-Transformer [35].

**Fig. 8:**
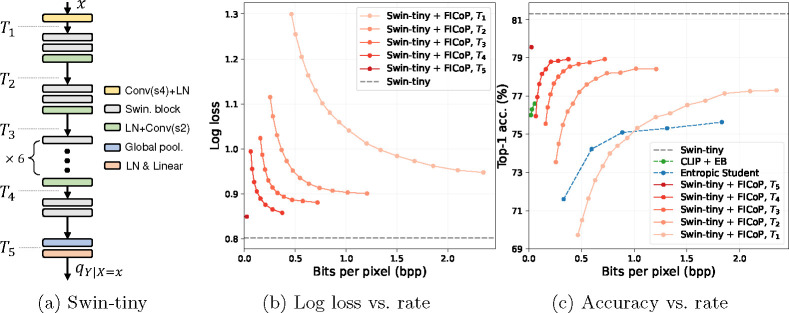
ImageNet classification using Swin-Transformer, *tiny* version (Swin-tiny) as the base model in terms of (b) distortion-rate and (c) accuracy-rate performance.

We also apply our approach to a Transformer architecture, Swin-Transformer, and show the results in Fig. 8. The observations are consistent with previous experiments, showing that our approach works well with Swin-Transformer and outperforms the baseline methods. We thus conclude that FICoP is a general approach that can be applied to various image classification model architectures.

### 5.4 Experimental analysis

We investigate our method by ablating its components. All other settings are the same as in the previous section.

#### Impact of joint training multiple entropy bottlenecks (EBs).

**Fig. 9:**
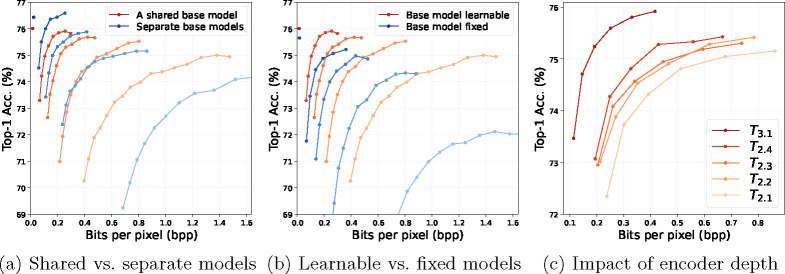
Experimental analysis of our approach on ImageNet. In (a) and (b), a lighter color represents a shallower layer, and the entropy bottleneck positions are the same as in Fig. 6a.

As our approach trains one shared base model jointly with multiple EBs, a natural question is how its performance differs from the one of training a separate base model for each EB. We thus train a separate ResNet-50 model for each EB at $\left\{T_{i.1},\;i=1,2,\ldots ,5\right\}$, and we refer to it as “separate base models” as shown in Fig. 9a. We observe that using a shared base model achieves comparable performance (better for low encoding complexity but worse for high encoding complexity) with multiple independent models while saving the parameters and training time by a factor of 5, as the latter needs five models in total.

#### A learnable base model is better than a fixed one.

Several prior methods also insert EBs into a pre-trained base model but keep the base model parameters fixed [1, 36, 37]. This approach has the advantage of allowing one to add new EBs incrementally without affecting the existing ones. We implement this strategy using our EBs and show the results in Fig. 9b. Compared to our joint training approach, it leads to worse accuracy-rate performance, especially in the low complexity regime, suggesting that it is important to finetune the base model such that it can adapt to the EBs.

#### Downsampling improves the accuracy-rate trade-off.

To understand how each ResNet-50 blocks contribute to the accuracy-rate performance, we place an entropy bottleneck after each block from $T_{2.1}$ to $T_{3.1}$ and show the results in Fig. 9c. The features at $\left\{T_{2.j},j=1,\;2,\;3,\;4\right\}$ have the same dimension, while the feature at $T_{3.1}$ is 2 times smaller due to downsampling in the ResNet block. We again observe that a deeper entropy bottleneck achieves a better accuracy-rate trade-off. Interestingly, the accuracy-rate curve of $T_{3.1}$ is much better than that of $\left\{T_{2.j},\;j=1,\;2,\;3,\;4\right\}$. This is presumably because downsampling removes the dimensions that contain the irrelevant information H(X∣Y) and thus can largely improve the rate-distortion performance.

## 6 Related Work

### Image/video coding for machines.

The most related works to our method are those on split computing [1, 36–39], where a pre-trained model is split into two parts and deployed on different devices, and the intermediate feature is compressed and then transmitted. However, to our knowledge, none of the existing works achieves end-to-end training of multiple splitting points as in ours. Several works considered compression for prediction under encoding complexity constraints [3, 40, 41], but the complementary relationship between encoder complexity and decoder complexity is not exploited. Other works design specific methods for particular applications such as video compression [42], privacy-preserving compression [4, 43], scalable coding [2, 44, 45], and the Internet of Things [6], which are orthogonal and can be combined with our method.

### Impact of computational constraints in data compression.

In applications such as video compression, it is widely known that the encoding computational complexity can largely impact the rate-distortion trade-off [46–48]. For example, in modern video codecs such as VVC [49], requiring a fast encoding speed can lead to up to more than 30% rate increase [50]. This is consistent with our observation in compression for prediction. Nevertheless, techniques developed for image/video compression cannot be directly applied to our case due to the different nature of the two problems.

### Information theory and compression in learning.

The objective of coding for machines resembles the one in information bottleneck methods [51–53], except that the latter does not require entropy coding. Thus, our study on the encoder/decoder complexity also applies to the information bottleneck methods. Several existing works also investigate the impact of computational constraints on information theory. Xu et al. [54] and Kleinman et al. [55] consider the amount of usable information between variables under constraints. A more related work is Harell et al. [10], where the authors prove that post-training compression of a deeper feature in a fixed model is better in terms of rate-distortion. Our paper explores the case where the base model is trainable, which is complementary to Harell et al. [10].

## 7 Conclusion

We have extended the rate-distortion trade-off in coding for machines to incorporate computational constraints. In particular, we show that a more powerful encoder leads to better rate-accuracy performance, and we reveal a complementary relationship between the required encoder size and the decoder size to achieve good performance. Experimental results have confirmed the existence of a three-way trade-off between rate, distortion, and model complexity, as well as showing the proposed method is advantageous over earlier methods in practical situations.

### Limitations and future work.

An important assumption in our method is that in the base model, the model’s input-feature-output forms a Markov chain, which is not true for models with hierarchical features and skip connections [56, 57]. Also, we only consider image classification as the downstream task in this work. Future work could investigate more general cases, e.g., machine vision tasks such as image segmentation, human vision tasks such as image denoising and inpainting, and a combination of both [58].

## Figures and Tables

**Table 1:** Encoding complexity of our method compared to previous ones. FLOPs are estimated for a 224 × 224 input image.

	ResNet-50 + FICoP (ours)					CLIP + EB [9]	Entropic Student [3]	ResNet-50
	*T*_1.1_	*T*_2.1_	*T*_3.1_	*T*_4.1_	*T*_5.1_			
Enc. params.	0.32M	1.02M	3.98M	17.8M	28.2M	87.9M	0.14M	25.6M
Enc. FLOPs	0.4G	1.2G	2.3G	3.7G	4.1G	4.4G	0.47G	4.1G

**Fig. B1:**
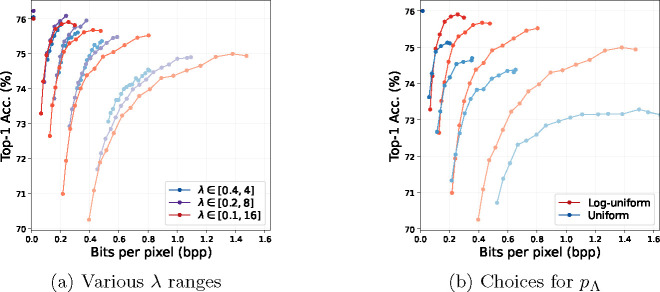
ImageNet classification experiments on the hyperparameters. The base model is ResNet-50. A deeper color indicates a deeper layer.

**Fig. B2:**
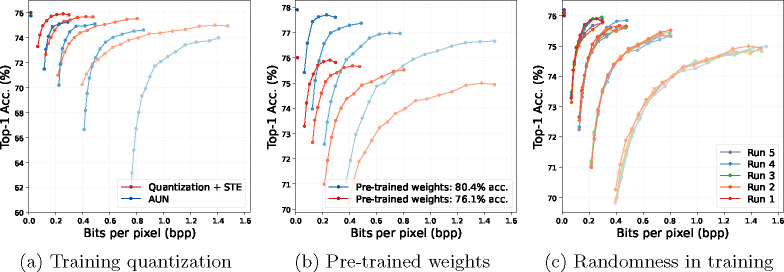
Additional experiments on ImageNet. The base model is ResNet-50. A deeper color indicates a deeper layer.

## Data Availability

The research presented in this manuscript relies on the ImageNet dataset, a well-known and publicly accessible database widely used in the field of computer vision. We will ensure that materials related to our research, including source code and trained models necessary for replicating the results reported, will be made publicly available upon the acceptance of the paper.

## Footnotes

^1^That said, the rate is dependent on the decoder during training, in the sense that the decoder affects the encoder parameters through backpropagation.

## Appendix A Training Hyperparameters

We show the ImageNet training hyperparameters in Table 1, which include three models: ResNet-50 [32], ConvNeXt [34], and Swin-Transformer [35]. For ResNet-50, we use the tv resnet50 model from the *timm* library^2^, which is pre-trained on ImageNet and has a top-1 accuracy of 76.1%. For ConvNeXt and Swin-Transformer, we use the models and training script provided by the *torchvision* library^3^.

**Table 1:** Imagenet training hyperparameters

	ResNet-50	ConvNeXt	Swin-Transformer

Train img. size	224 × 224	176 × 176	224 × 224
Val img. resize	236 × 236	236 × 236	224 × 224
Val img. crop	224 × 224	224 × 224	224 × 224
Interpolation	Bi-linear	Bi-linear	Bi-cubic

Data augment.	Crop, h-flip	TrivialAugment [59].	TrivialAugment [59].
Random erasing	-	0.1	0.25
Label smoothing	-	0.1	0.1
Mix-up	-	0.2	0.8
Cut-mix	-	1.0	1.0

Optimizer	AdamW	AdamW	AdamW
Learning rate	1e-4	1e-4	1e-4
LR warm-up	Linear, 8k iters	Linear, 5 epochs	Linear, 20 epochs
LR schedule	Cosine	Cosine	Cosine
Weight decay	0.05	0.05	0.05
Gradient clip	-	-	5.0

Batch size	256	256	256
# iterations	160k	160k	160k
# images seen	41M	41M	41M
# epochs	32	32M	32

EMA	0.9999	0.99998	0.99998

GPU type	Quadro RTX 6000	RTX 3090	RTX 3090
GPU num	2	2	2
Training time	18 hours	9 hours	16 hours

The training devices and the training time for each experiment are also reported in Table 1. We estimate the total computation for all experiments is around 800 GPU hours (more than 20 training runs for ResNet-50, each one costing around 36 GPU hours).

## Appendix B Additional Experiments

In this section, we provide additional experimental analysis to further analyze our method as well as support our claims in the main paper. We use ResNet-50 as the base model for ImageNet classification, and unless otherwise specified, we use the same hyperparameters as in the main paper.

### B.1 Choice of the hyperparameters

#### The range of λ.

We vary the range of λ used during training to see its impact on the R-D performance. In addition to the default range λ∈[0.1,16], we tried two ranges: λ∈[0.4,4] and λ∈[0.2,8]. The results are shown in Fig. B1a. We first observe that a larger range of λ leads to a wider range of the rate-distortion curve, which is expected since λ controls the rate-distortion trade-off. In addition, reducing the λ range results in a slightly better rate-distortion performance, which is also expected, as the model only needs to focus on a smaller range of rates.

#### Choices of $p_{\Lambda }$.

The distribution of λ used during training can be freely chosen by the user. Here, we compare the impact of different choices of $p_{\Lambda }$. Our default choice is a log-uniform distribution, i.e., $log\Lambda ∼U\left(\log\lambda_{\text{low}},log\lambda_{\text{high}}\right)$, where $\lambda_{\text{low}}$ and $\lambda_{\text{high}}$ are the lower and upper bounds of λ. This distribution is commonly used for hyperparameter searching. We also tried a uniform distribution, i.e., $\Lambda ∼U\left(\lambda_{\text{low}},\lambda_{\text{high}}\right)$. The comparison is shown in Fig. B1b. We observe that the log-uniform distribution leads to a clearly better accuracy-rate performance than the uniform distribution, especially at high rates.

### B.2 Ablatve analysis

#### Quantization.

As stated in Sec. 4.2, we apply hard quantization to the latent variables, and the gradients are approximated by the straight-through estimator (STE). Contrary to many learning-based image compression methods, we do not use additive uniform noise (AUN). We show the comparison between these two strategies in terms of accuracy-rate performance in Fig. B2a. The results show that quantization with STE clearly outperforms AUN, which leads to the choice of STE in our method.

#### Better pre-trained weights improve performance.

We compare the impact of different pre-trained weights. In the main paper, we use the pre-trained weights that achieve 76.1% top-1 accuracy on ImageNet, which is the standard pre-trained weights used in literature. We also try the pre-trained weights provided by the *timm* library that achieve 80.4% top-1 accuracy on ImageNet. The results are shown in Fig. B2b, where we observe that the better pre-trained weights lead to clearly better accuracy-rate performance, especially at deeper layers.

#### Impact of random seed.

To study the impact of randomness in training, we train FICoP 5 times with different random seeds. The results for these runs are shown in Fig. B2c. We observe that although the performance varies slightly depending on the random seed, the overall trend is consistent. It is interesting to note that if we compare the leftmost and rightmost edges of each curve, we can observe that the rate does not vary much across different runs, while the accuracy varies more.
